# Improved and Novel Methods for Investigating Organophosphate Esters in Particulate Matter

**DOI:** 10.3390/analytica5040032

**Published:** 2024-10-02

**Authors:** Annie Gathof, Tess Bonanno, Paige Rossicone, Adelaide E. Clark

**Affiliations:** Department of Chemistry and Biochemistry, Providence College, One Cunningham Square, Providence, RI 02918, USA

**Keywords:** pressurized liquid extraction, ASE, particulate matter, organophosphate ester, OPE, flame retardant, plasticizer, extraction method

## Abstract

A pressurized liquid extraction (PLE) method for the extraction of 31 organophosphate esters (OPEs) and novel organophosphate esters (NOPEs) has been developed. Unlike previously published methods, this method utilizes the high-throughput nature of PLE (as opposed to Soxhlet or sonication methods) without using potentially harmful organic solvents like methylene chloride. Combinations of hexane and acetone and hexane and ethyl acetate at various temperatures were examined. Extracts were concentrated and analyzed via gas chromatography–mass spectrometry. The final optimized method utilized 1:1 *v*/*v* hexane/ethyl acetate at 100 °C for three static cycles (5 min each) at 80% flush volume and a 100 s N_2_ purge. This provided average surrogate corrected target analyte percent recoveries in spike and recovery experiments (*n* = 6) for OPEs and NOPEs of 106 ± 13%, with average surrogate recoveries of 88.6 ± 7.3%. The developed method was further validated using standard reference materials and was then applied to atmospheric particulate matter samples collected in the city of Providence, RI. The dataset reflected ambient concentrations of 16 OPEs and NOPEs (reported in pg m^−3^) for the first time in the greater Providence metropolitan area, including one of the first reports of NOPEs in atmospheric particulate matter in the U.S.

## 1. Introduction

Atmospheric particulate matter (PM) is an efficient medium for the sorption of organic chemicals, making it an effective indicator of pollution in the atmosphere [1]. Air pollution remains “one of the biggest environmental threats to human health”, according to the World Health Organization (WHO) [2], making the health effects associated with these chemicals a hazard of air pollution [3].

Organophosphate esters (OPEs) are “high production-volume” chemicals (>1000 tons annually) [4,5] which are a “reemerging” pollutant with a wide range of uses in consumer products, including as a flame retardant (FR) and plasticizer [6–11]. OPEs were first reported in the environment in the 1970s [12], but studies of them were all but abandoned in the 1990s as alkyl- and aryl-substituted compounds were believed to be degradable in the environment [7]. The expectation that OPEs would be less persistent and would not accumulate in the environment led to their widespread use in recent years [13,14], in part as a replacement FR for polybrominated diphenyl ethers (PBDEs), but this expectation was contradicted by high concentrations detected in remote regions [15,16].

With the restriction of “traditional” OPEs in certain markets [5], there has been a demand for new OPE alternatives [8,17]. These “novel” OPEs (NOPEs), as they have been termed, have been added to some target analyte lists in studies of PM [18,19], but most extraction methods have not been updated or expanded to include these NOPEs.

Pressurized liquid extraction (PLE), introduced in 1996, improved the extraction of environmental samples over more traditional Soxhlet or sonication techniques by decreasing extraction time and reducing the volume of organic solvents used [20,21]. Despite these improvements, many studies on OPEs in PM still rely on Soxhlet [22–25] or sonication [2,18,19,22,26] extraction methods for sample analysis. Additionally, both PLE methods and non-PLE methods for extraction continue to use methylene chloride (DCM) as an extraction solvent [2,22,24,27,28] despite ongoing concerns about human and environmental health with prolonged exposures and with recent use bans in the United States [29,30].

The goal of this study was to develop a PLE method for the extraction of OPEs (and other markers of urban pollution) from filter-based atmospheric PM samples that does not rely upon potentially hazardous solvents like DCM. This analytical method was validated, in part, using Standard Reference Material (SRM) 2585, and was then used to examine filter-based samples taken as part of routine monitoring in an urban area (Providence, RI, USA).

## 2. Materials and Methods

All solvents, analytical standards, and other materials were obtained from commercially available vendors. DCM and acetone (ACE) were purchased at 99.5% purity, while hexanes (HEX) and ethyl acetate (EA) were purchased at 99.4% and 99.6% purity, respectively (Avantor, Center Valley, PA, USA, distributed by VWR, Radnor, PA, USA). Specific vendors for each standard and compound name abbreviations can be found in Table S1. Quartz fiber filters (QFF; 20 × 25 cm) were purchased from Pall Life Sciences (Port Washington, NY, USA; distributed by VWR, Radnor, PA, USA). Standard Reference Material (SRM) 2585 (Organic Contaminants in House Dust) was purchased from the National Institute of Standards and Technology (Gaithersburg, MD, USA, distributed by Millipore Sigma, Burlington, MA, USA).

QFFs were prepared by wrapping in aluminum foil, baking at 500 °C for at least 12 h, and then storing at −20 °C, as described previously [27]. Prepared filters were used in all experiments described below, including as blank samples, to measure possible laboratory contamination during sample preparation.

To optimize pressurized liquid extraction (PLE), parameters such as solvent composition and extraction temperature were independently varied. All PLE was carried out using an Accelerated Solvent Extractor system (ASE; 350 System, Thermo Scientific^™^ Dionex^™^, Waltham, MA, USA). Overall extraction efficiencies were assessed based on surrogate standard and target analyte percent recoveries, which were compared using ANOVA and Student’s *t*-tests (Analysis Toolpak, Microsoft Excel, Microsoft 365 v. 16). The suitability of extracts for chemical analysis was also taken into consideration. ASE extraction cells (34 mL) and all other glassware were pre-cleaned as previously described [27]. Extracts were collected in pre-rinsed 200 mL collection bottles with ultra-clean septa (Thermo Scientific^™^ Dionex^™^, Waltham, MA, USA).

Once extracted, samples were concentrated using a Biotage Turbovap II (Biotage, Charlotte, NC, USA) at 35 °C under a gentle stream of N_2_. Rinses were performed periodically using HEX. Samples were then transferred to pre-rinsed amber GC vials once volumes were less than 500 μL, and a known amount of the isotopically labeled internal standard (d_12_-benzo(e)pyrene) was added prior to chemical analysis.

Chemical analysis was carried out using a Shimadzu GCMS-QP2020 NX utilizing electron ionization (EI), an SH-I-5MS capillary column (30 m × 0.25 mm i.d.; 0.25 μm film thickness) and helium carrier gas (99.999%) at a 0.69 mL min^−1^ flow rate. The previously published method [27] was optimized to ensure good chromatographic separation while decreasing the overall runtime from 54.00 min to 48.00 min. Briefly, 1 μL of the extract was injected in splitless mode at 280 °C. The temperature program for chromatographic separation was as follows: 100 °C held for 6 min, then ramped at 10 °C min^−1^ to 260 °C, then ramped at 4 °C min^−1^ to 300 °C and held for 5 min, and then ramped at 20 °C min^−1^ to 320 °C and held for 10 min for a total runtime of 48.00 min. The MS interface was held at 280 °C, and the ion source was set at 230 °C. Table S1 summarizes the approximate retention times as well as the ions used for quantitative and qualitative analysis. Selective ion monitoring was used for all identifications.

The identification of analytes was achieved via retention time (±0.05 min) and ion ratios (±20%). Analytes were quantitated via 7–8 point calibration curves spanning 2–3 orders of magnitude and containing isotopically labeled surrogate and internal standards. Calibration curves had a minimum R^2^ of 0.99. The linear range of the calibration curve and R^2^ value for each compound is summarized in Table S1. Surrogate standards were those spiked in known amounts prior to extraction, while internal standards were those spiked in known amounts prior to GC-MS analysis. Target analytes were quantified and corrected to surrogates to account for losses during the sample preparation process, while surrogates were quantified and corrected to internal standards to account for day-to-day GC-MS variability, as previously described [27]. Samples were run on the GC-MS in batches of 3–6 with a mid-point calibration check standard (CCV) run before and after each batch to ensure calibration validity. Solvent blanks (DCM) were run before and after each CCV.

To evaluate the reproducibility, precision, and accuracy of the final methodology, six blank QFF aliquots (the maximum size of a real sample batch) were spiked with known amounts of target analytes and surrogate standards. A seventh QFF aliquot was spiked with surrogate standards only to serve as a laboratory blank for correction purposes. Once spiked, samples were allowed to come to a quasi-equilibrium for 90 min prior to extraction. These spiked samples were taken through the final analytical method, and a known amount of isotopically labeled internal standard was spiked prior to chemical analysis. The percent recovery (±standard deviation) for each surrogate and surrogate-corrected target analyte was calculated to help evaluate the precision of the method. An additional reproducibility study (*n* = 3) was performed on a separate day by a separate analyst using the optimized analytical method. Percent recoveries were again calculated and compared via paired *t*-tests to determine any statistical difference.

To further evaluate the accuracy of the final method, SRM 2585 (Organic Contaminants in House Dust) was analyzed. A precleaned aliquot of QFF was loaded with 55–85 mg of dust, spiked only with known amounts of surrogate standards (for quantitation), and taken through the final analytical method. Following extract concentration, known amounts of isotopically labeled internal standards were added before chemical analysis. The percent errors between detected values and reported certified and reference values were calculated by subtracting the detected value from the reported value and then dividing by the reported value.

Estimated method detection limits (MDLs) were determined via EPA protocols [31]. Briefly, seven aliquots of QFF were spiked with target analytes at levels between 2 and 10 times the expected MDLs and with surrogate standards. An additional seven blank aliquots were spiked with just surrogate standards. These 14 samples were broken into three separate batches (4–6 samples each, at least 2 spiked and at least 2 blanks), and extracted and analyzed (using the final analytical method) over three separate calendar days. EPA protocols for the mathematical determination of MDLs were used, and the higher of the two calculated MDLs (spiked and blank) was used as the MDL for each analyte.

Atmospheric PM samples were collected as part of Friar Air Monitoring Network (FriAir Net) routine monitoring at Providence College (PC; 41.842716, −71.438802) and near the Port of Providence (41.795139, −71.397906) using a Tisch Total Suspended Particle (TSP) sampler TE-PNY1123 (Tisch Environmental, Cleves, OH, USA). Five samples each for TSP were collected on a 1-in-6-day sampling schedule in February and March 2024. Samples were collected for 24 h beginning at 6:00 am (6:05 am for PM_2.5_) on the sample date and ending at 6:00 am (6:05 am for PM_2.5_) on the following morning.

Filter aliquots representing 200 cm^2^ of QFF were placed in 34 mL ASE cells and spiked with known amounts of isotopically labeled surrogate standards and processed through the final analytical method. Samples were batched by location. A lab-blank was included in each batch of samples, and samples were blank-corrected as needed. Concentrated extracts were spiked with known amounts of the isotopically labeled internal standard prior to chemical analysis. Concentrations of target analytes in the samples were normalized to the ambient volume of air each filter sampled as calculated by the sampler based on runtime, flow rate, and ambient conditions.

## 3. Results and Discussion

### 3.1. Optimization

A total of eight experiments (Table 1) were conducted to determine the optimal extraction solvent combination and temperature. A ninth experiment using the previously published 2:1 DCM/ACE at 100 °C was also run for comparison [27]. Although the variables in PLE that affect the efficiency of the extraction also include extraction pressure, the number of extraction cycles, and length of extraction cycles, the temperature and type of solvent appear to have the greatest impact on extraction efficiency [32,33] and were, therefore, the focused variables in this study. Surrogate recoveries were used to assess the efficiencies of various solvent combinations and extraction temperatures and were compared via ANOVA and Student’s *t*-test. To begin and eliminate the need for DCM as an extraction solvent, HEX and ACE were combined at varying ratios (1:1 vs. 2:1) and temperatures (100 °C vs. 120 °C). Average surrogate standard recoveries (Table 1) for experiments 2–5 had no statistically significant difference with experiment 1, with the exception of experiment 2, which was significantly higher. However, extracts were inconsistently compromised by the presence of water, forming an immiscible layer during the final concentration step before chemical analysis.

To eliminate this inconsistency while retaining similar polar properties, EA was substituted for ACE in experiments 6–9. With the exception of experiment 8, which was significantly lower, there was no statistically significant difference between these experiments and experiment 1. This suggests that a solvent combination of HEX/EA is comparable to 2:1 DCM/ACE for OPEs while eliminating the need to use a chlorinated solvent in the extraction. Experiment 6 (1:1 *v*/*v* HEX/EA at 100 °C) proved to be statistically significantly higher than experiments 7–9, with an average surrogate recovery of 73.8 ± 8.1%. For this reason, experiment 6 was chosen as the final methodology, utilizing 1:1 *v*/*v* HEX/EA at 100 °C with three static cycles, each 5 min long, with an 80% flush volume and a 100 s N_2_ purge. Following each extraction, the ASE performed a rinse using HEX.

### 3.2. Validation

#### 3.2.1. Reproducibility Studies, SRM, and MDLs

**Figure 1.**
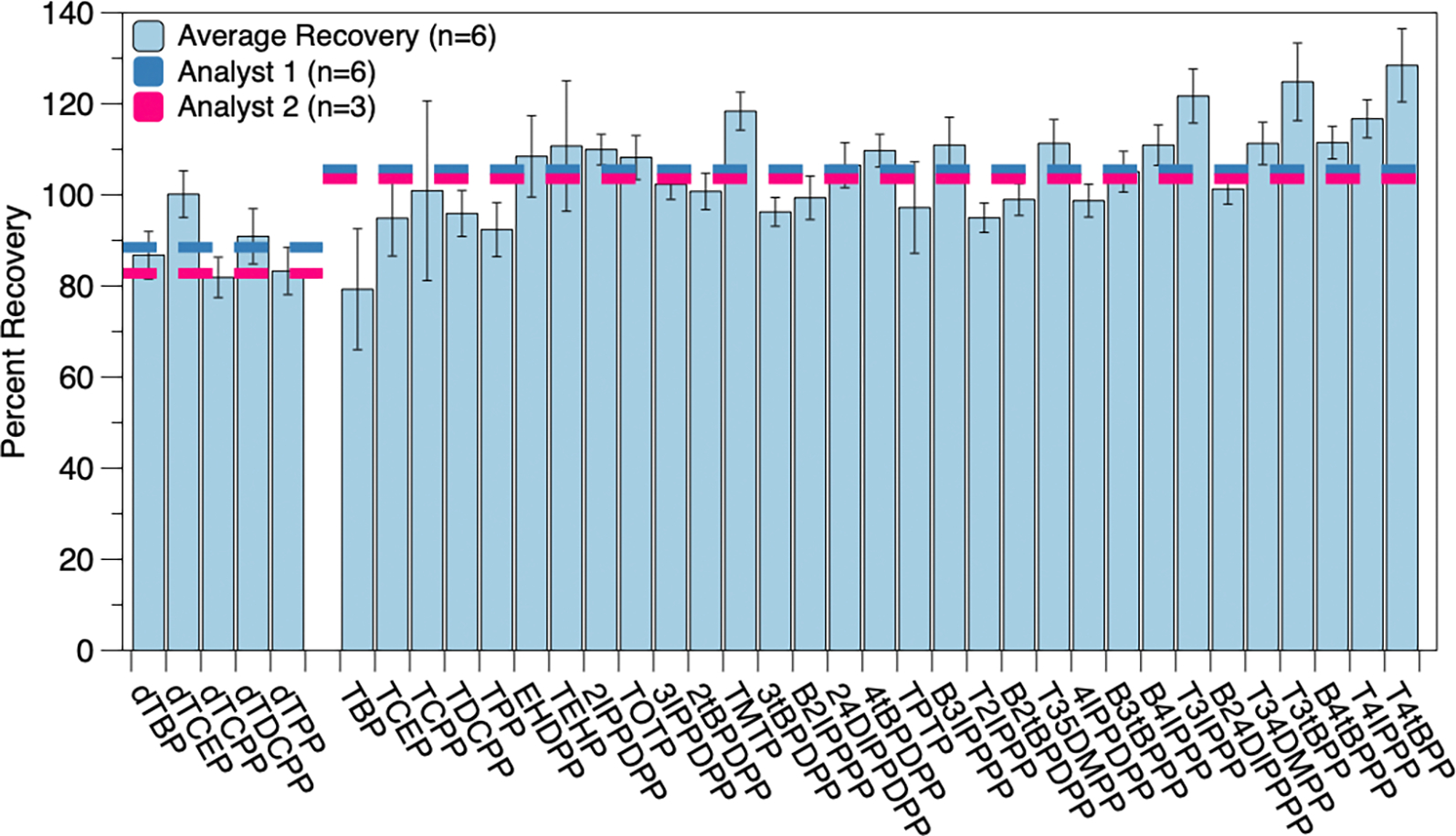
Average recoveries for OPEs from Analyst 1^′^s reproducibility study (*n* = 6) for the final analytical method. The left set of blue bars represents average surrogate recoveries, while the right set of blue bars indicates surrogate- and blank-corrected target analyte recoveries. The overall average recoveries for this study are indicated by the dark blue dashed line. The overall average surrogate and target analyte recoveries from Analyst 2^′^s reproducibility study (*n* = 3) are indicated by the dashed pink-like. Compound names and abbreviations are given in Table S1.

The chosen methodology was further validated for accuracy and precision by conducting reproducibility studies and analyzing an SRM, as described above. For the reproducibility study, six replicates (simulating the largest size for a batch of actual environmental samples) were spiked prior to extraction and provided a range of average surrogate recoveries from 81.9 ± 4.4% to 100 ± 5%. The average surrogate-corrected and blank-corrected target analyte recovery was 106 ± 13%. Individual recoveries can be seen in Figure 1 and are summarized in Table 2. An additional reproducibility study (n = 3) was carried out by separate laboratory personnel (Figure 1). Surrogate recoveries for this study ranged from 74.2 ± 1.9% to 94.3 ± 4.5%, with average surrogate corrected target analyte recoveries of 104 ± 10%. These recoveries are similar to those previously reported [27], even with the expanded target analyte list represented in this study, and were not statistically different from one another.

To further evaluate accuracy and precision, three replicates of the SRM were analyzed as outlined above. Three of the four target OPEs which have reference values in SRM 2585 were detected within 10% or less of the reported values. It is worth noting that the fourth (tri-*n*-butyl phosphate, TBP) was near the MDL at the mass used for this analysis, which could explain the larger percent error observed. An additional 13 OPEs/NOPEs were detected in SRM 2585, which do not have values in the certificate of analysis (Table 2). MDLs were determined, as outlined above, and ranged from 14.2 to 55.9 ppb. Individual MDLs for each compound are given in Table 2.

#### 3.2.2. Environmental Samples

**Figure 2.**
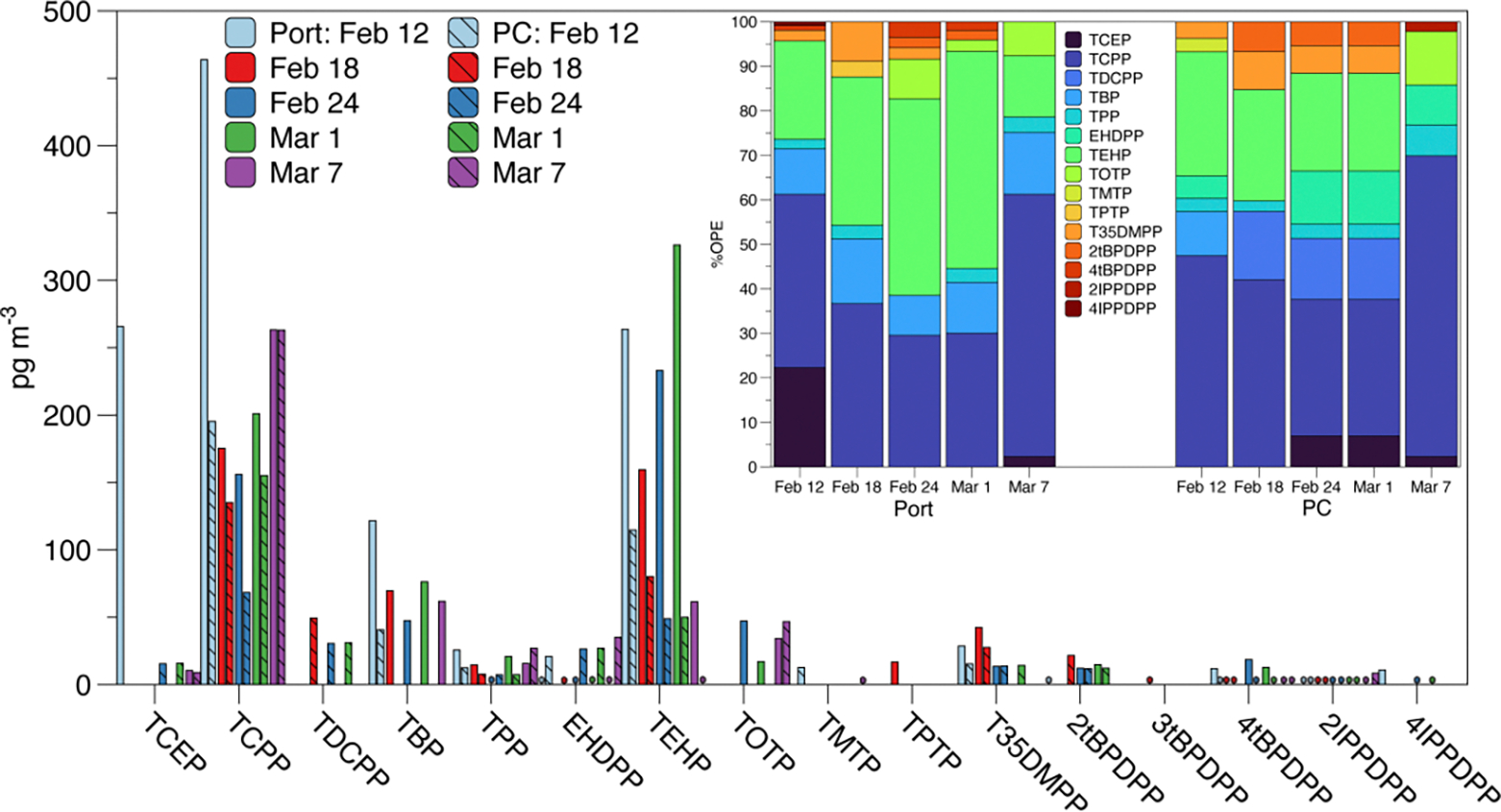
Atmospheric particulate matter concentrations of detected OPEs in samples taken at the Port of Providence (solid bars) and Providence College (PC; striped bars). * indicate < MDL. Inlay shows percent composition of ΣOPE in each sample for each compound detected above MDL. Compound names and abbreviations are given in Table S1.

OPEs and NOPEs were detected in all five samples from both sampling sites (Figure 2). Tris[(2R)-1-chloro-2-propyl] phosphate (TCPP), triphenyl phosphate (TPP), tris(2-ethylhexyl) phosphate (TEHP), 2-isopropylphenyl diphenyl phosphate (2IPPDPP), and 4-tert-butylphenyl diphenyl phosphate (4tBPDPP) were consistently detected in every sample from both sites. Additionally, TBP was detected in all the samples from the Port, and 2-ethylhexyl diphenyl phosphate (EHDPP) was detected in all the samples from PC. It is worth noting that while detected in every sample, 2IPPDPP was consistently <MDL. Most of the detected atmospheric concentrations of OPEs and NOPEs were higher in samples from the Port compared to PC, with the exception of EHDPP and tris(1, 3-dichloro-2-propyl) phosphate (TDCPP), which was higher at PC. This suggests a closer source proximity or prominence of sources at the Port compared to PC. The percent composition of ΣOPE (Figure 2 inset) also suggests a more diverse source of OPEs and NOPEs at the Port compared to PC. Additionally, the predominance of chlorinated OPEs at PC agrees with previous studies in other United States (U.S.) urban/suburban areas [23,34], though ΣOPE (695 ± 306 pg m^−3^ at the Port; 342 ± 96 pg m^−3^ at PC) is lower than previous reported urban concentrations. This represents one of the first U.S.-based studies on atmospheric concentrations of OPEs in PM in over a decade and one of the first report of NOPEs in U.S. PM. As such, this bears further analysis and will be investigated utilizing the extraction methodology described here.

### 3.3. Additional Considerations (PAHs)

While the focus of this study is on the extraction of OPEs from filter-based particulate matter samples, the extraction method was also optimized and validated for 13 PAHs (Tables S2 and S3). Surrogate recovery data for PAHs are included in the optimization results and discussion above and contributed to the choice of the final method parameters (1:1 *v*/*v* HEX/EA at 100 °C). Full validation data for PAHs are included in the Supplementary Information (Figures S1 and S2). Similarly to the OPEs, PAHs showed good surrogate recovery in reproducibility studies (70.3 ± 8.3% and 68.2 ± 6.6% for *n* = 6 and *n* = 3, respectively) with average target analyte recoveries of 103 ± 5% and 99.2 ± 6.5%, respectively (Figure S1). SRM percent errors ranged from 3 to 49%, with the exception of anthracene, which was near the method detection limits and produced large variations between samples (Table S3). Finally, all 13 PAHs were detected in samples from both sites, showing consistent ratios of compounds to each other (Figure S2), which suggests robust and constant sources of PAHs to these sites, as expected due to the urban location and proximity to the Interstate 95 corridor and other industries in the area.

## 4. Conclusions

This report describes a method developed for the extraction of 31 OPEs and NOPEs from filter-based atmospheric particulate matter samples. This method builds upon methods previously developed by expanding the target analyte list to include NOPEs while also utilizing high-throughput techniques (PLE) and eliminating the use of potentially harmful chlorinated solvents (DCM). The final methodology utilized a solvent composition of 1:1 *v*/*v* HEX/EA, run at 100 °C for three static cycles, 5 min long each with, an 80% flush volume and 100 s N_2_ purge.

The final methodology was shown to be robust and precise, with two analysts evaluating six and three replicates, respectively. The average recoveries (106 ± 13% and 104 ± 10%, respectively) for the 31 target analytes were similar to those found using previously published PLE methodology and were not statistically different from each other. Method accuracy was also evaluated using SRM 2585, and low percent differences with reported values (10% or less) confirmed the accuracy of the method.

Finally, the method was used to analyze TSP samples taken from two sampling sites in Providence, RI. In total, 16 OPEs and NOPEs were detected between the two sites, with five compounds detected in all samples from both sites. This is one of the first U.S.-based atmospheric concentrations of OPEs in PM reported in over a decade, with the first reported in the state of Rhode Island and one of the first U.S.-based atmospheric concentrations of NOPEs in PM reported. The use of this method will allow for high-throughput extraction, aiding the analysis of large sample sets while also eliminating the use of chlorinated solvents. This will allow for further measurements of OPEs and NOPES in atmospheric particulate matter samples taken from the U.S. and around the globe, allowing for a better understanding of these compounds in the environment.

## Supplementary Material

Supplementary Material

## Figures and Tables

**Table 1.** List of experiments undertaken to choose final methodology with average surrogate recoveries ± one standard deviation.

Exp. #	Solvent Composition	Extraction Temp. (°C)	Surrogate Recovery ^1^ (Average ± Standard Deviation) (%)

1	2:1 DCM/ACE	100	71.1 ± 23.1 ^2^
2	1:1 HEX/ACE	100	88.1 ± 18.8 ^2^
3	1:1 HEX/ACE	120	74.4 ± 18.1 ^2^
4	2:1 HEX/ACE	100	56.0 ± 10.8 ^2^
5	2:1 HEX/ACE	120	66.5 ± 19.2 ^2^
**6**	**1:1 HEX/EA**	**100**	**73.8 ± 8.1** ^3^
7	1:1 HEX/EA	120	64.6 ± 8.6 ^3^
8	2:1 HEX/EA	100	56.0 ± 8.4 ^3^
9	2:1 HEX/EA	120	62.4 ± 10.3 ^3^

1 Reported surrogate recoveries include recoveries for PAH surrogates. See “Section 3.3 Additional Considerations” below.

2 Recoveries include two OPE surrogates, dTCEP and dTPP.

3 Recoveries include final five OPE surrogates.

**Table 2.** Method validation delta for each OPE and OPE surrogate, including; percent recovery from reproducibility study, method detection limit (MDL), and detected versus reported amounts from SRM 2585 with calcula tea percent error. Compound abbreviations are given in Table S1.

	Precision and Accuracy		SRM 2585		

Compound Abbr.	Percent Recovery (*n* = 6; %)	MDL (ppb)	Detected (pg mg^−1^)	Reported (pg mg^−1^)	% Error

dTBP	86.8 ± 5.2	---	---	---	---
TBP	79.3 ± 13.3	51.0	437 ± 64	276 ± 14	58%
dTCEP	100 ± 5	---	---	---	---
TCEP	94.9 ± 8.3	14.2	872 ± 21	925 ± 149	6%
dTCPP	81.9 ± 4.4	---	---	---	---
TCPP	101 ± 20	41.1	1157 ± 250	1220 ± 350	5%
dTDCPP	90.9 ± 6.1	---	---	---	---
TDCPP	95.9 ± 5.1	39.7	3149 ± 238	NR^1^	---
dTPP	83.3 ± 5.2	---	---	---	---
TPP	92.4 ± 5.9	53.4	1282 ± 105	1190 ± 130	8%
EHDPP	108 ± 9	34.3	1086 ± 57	NR ^1^	---
TEHP	111 ± 14	55.9	939 ± 48	NR ^1^	---
2IPPDPP	110 ± 3	26.7	431 ± 57	NR^1^	---
TOTP	108 ± 5	27.4	139 ± 47	NR^1^	---
3IPPDPP	102 ± 3	24.0	---	---	---
2tBPDPP	101 ± 4	19.0	---	---	---
TMTP	118 ± 4	20.9	---	---	---
3tBPDPP	96.3 ± 3.2	19.2	97.6 ± 11.2	NR ^1^	---
B2IPPPP	99.4 ± 4.8	14.4	231 ± 30	NR ^1^	---
24DIPPDPP	107 ± 5	18.2	314 ± 64	NR ^1^	---
4tBPDPP	110 ± 4	19.6	481 ± 29	NR ^1^	---
TPTP	97.2 ± 10.0	16.1	239 ± 8	NR ^1^	---
B3IPPPP	111 ± 6	25.3	---	---	---
T2IPPP	95.0 ± 3.2	19.4	174 ± 13	NR ^1^	---
B2tBPPP	99.0 ± 3.5	17.7	---	---	---
T35DMPP	111 ± 5	21.9	---	---	---
4IPPDPP	98.7 ± 3.6	17.4	---	---	---
B3tBPPP	105 ± 4	22.0	---	---	---
B4IPPP	111 ± 4	21.7	---	---	---
T3IPPP	122 ± 6	24.5	---	---	---
B24DIPPPP	101 ± 3	21.5	---	---	---
T34DMPP	111 ± 5	17.6	---	---	---
T3tBPP	125 ± 9	18.2	---	---	---
B4tBPPP	111 ± 4	22.9	294 ± 24	NR ^1^	---
T4IPPP	117 ± 4	20.2	---	---	---
T4tBPP	128 ± 8	25.9	113 ± 19	NR ^1^	---

1 No values for these compounds are given in the NIST certificate of analysis for SRM 2585.

## Data Availability

Data are available upon request from the authors.
