# Supplementary material for: Improved and Novel Methods for Investigating Organophosphate Esters in Particulate Matter
Source: Analytica (Basel). Author manuscript; Available in PMC 2025 Nov 18. (PMC12621544; doi:10.3390/analytica5040032)
Supplement: Supplementary Material [file NIHMS2057623-supplement-Supplementary_Material.pdf]

Supplemental Information for: Improved and Novel Methods for Investigating Organophosphate Esters in Particulate Matter

Annie Gathof, Tess Bonanno, Paige Rossicone and Adelaide E. Clark *

Department of Chemistry and Biochemistry, Providence College, One Cunningham Square, Providence, RI 02918, USA
* Correspondence: aclark6@providence.edu

Table of Contents

Table S1 2
Table S2 4
Table S3 5
Figure S1 6
Figure S2 6

Table S1. List of OPEs names and abbreviations as well as data for GC-MS analysis such as retention time, ions monitored, surrogate standards used for quantitation and calibration information. Footnote on compound name indicates standard manufacturer.

Compound Name	Abbr.	Approx. Retention Time (min)	Quantitative Ion	Qualitative Ions	Quantitation Standard	Linear Range (pg µL^{-1})	R^2
d_{27}-Tri-n-butyl phosphate[1]	dTBP	15.9	103	167, 231	dBeP	---	---
Tri-n-butyl phosphate[2]	TBP	16.1	99	155, 211	dTBP	3.4-1140	0.997
d_{12}-Tris(2-chloroethyl) phosphate[2]	dTCEP	17.5	261	148, 263	dBeP	---	---
Tris(2-chloroethyl) phosphate[2]	TCEP	17.7	249	99, 143	dTCEP	3.4-1140	0.999
d_{18}-Tris[(2R)-1-chloro-2-propyl] phosphate[1]	dTCPP	17.8	103	131, 293	dBeP	---	---
Tris[(2R)-1-chloro-2-propyl] phosphate[2]	TCPP	18.0	125	157, 293	dTCPP	3.4-1140	0.999
d_{15}-Tris(1, 3-dichloro-2-propyl) phosphate[1]	dTDCPP	23.4	79	103, 197	dBeP	---	---
Tris(1, 3-dichloro-2-propyl) phosphate[2]	TDCPP	23.6	99	191, 379	dTDCPP	3.4-1140	0.999
d_{15}-Triphenyl phosphate[2]	dTPP	24.3	341	243, 340	dBeP	---	---
Triphenyl phosphate[2]	TPP	24.4	326	169, 215	dTPP	3.4-1140	0.999
2-Ethylhexyl diphenyl phosphate[2]	EHDPP	24.6	251	55 94	dTPP	3.4-1140	0.999
Tris(2-ethylhexyl) phosphate[2]	TEHP	24.7	99	113	dTPP	3.4-1140	0.999
2-Isopropylphenyl diphenyl phosphate[2]	2IPPDPP	25.8	118	251, 368	dTPP	3.4-1140	0.999
Tri-o-tolyl-phosphate[2]	TOTP	26.4	368	169, 228	dTPP	3.4-1140	0.999
3-Isopropylphenyl diphenyl phosphate[2]	3IPPDPP	26.7	353	118, 368	dTPP	3.4-1140	0.999
2-tert-Butylphenyl diphenyl phosphate[2]	2tBPDPP	26.9	367	115, 382	dTPP	3.4-1140	0.999
Tri-m-tolyl-phosphate[2]	TMTP	27.2	368	91, 165	dTPP	3.4-1140	0.999
3-tert-butylphenyl diphenyl phosphate[2]	3tBPDPP	27.3	367	382	dTPP	3.4-1140	0.998
Bis(2-isopropylphenyl) phenyl phosphate[2]	B2IPPPP	27.2	118	215, 410	dTPP	3.4-1140	0.999
2,4-Diisopropylphenyl diphenyl phosphate[2]	24DIPPDPP	28.2	145	160, 410	dTPP	3.4-1140	0.999
4-tert-Butylphenyl diphenyl phosphate[2]	4tBPDPP	28.3	367	77, 382	dTPP	3.4-1140	0.999
Tri-p-tolyl phosphate[2]	TPTP	28.4	368	107, 261	dTPP	3.4-1140	0.999
Bis(3-isopropylphenyl) phenyl phosphate[2]	B3IPPPP	28.0	410	251, 395	dTPP	3.4-1140	0.999
Tris(2-isopropylphenyl) phosphate[2]	T2IPPP	28.3	367	77, 382	dTPP	3.4-1140	0.999
Bis(2-tert-butylphenyl) phenyl phosphate[2]	B2tBPPP	30.6	423	91, 367	dTPP	3.4-1140	0.999
Tris(3,5-dimethylphenyl) phosphate[2]	T35DMPP	30.7	410	194	dTPP	3.4-1140	0.999
4-isopropylphenyl diphenyl phosphate[2]	4IPPDPP	27.4	353	77	dTPP	3.4-1140	0.999
Bis(3-tert-butylphenyl) phenyl phosphate[2]	B3tBPPP	30.3	367	423, 438	dTPP	3.4-1140	0.999
Bis(4-isopropylphenyl) phenyl phosphate[2]	B4IPPP	30.7	395	190, 410	dTPP	3.4-1140	0.999
Tris(3-isopropylphenyl) phosphate[2]	T3IPPP	31.4	452	91, 118	dTPP	3.4-1140	0.999
Bis(2,4-diisopropylphenyl) phenyl phosphate[2]	B24DIPPPP	31.6	160	145, 494	dTPP	3.4-1140	0.999

Compound Name	Abbr.	Approx. Retention Time (min)	Quantitative Ion	Qualitative Ions	Quantitation Standard	Linear Range (pg μL^{-1})	R^2
Tris(3,4-dimethylphenyl) phosphate[2]	T34DMPP	33.0	410	193	dTPP	3.4-1140	0.999
Tris(3-tert-butylphenyl) phosphate[2]	T3tBPP	33.4	479	367, 423	dTPP	3.4-1140	0.998
Bis(4-tert-butylphenyl) phenyl phosphate[2]	B4tBPPP	32.6	423	367, 438	dTPP	3.4-1140	0.999
Tris(4-isopropylphenyl) phosphate[2]	T4IPPP	34.4	437	395, 452	dTPP	3.4-1140	0.999
Tris(4-tert-butylphenyl) phosphate[2]	T4tBPP	38.1	479	232, 367	dTPP	3.4-1140	0.997

[1] Purchased from Cambridge Isotope Laboratories (Andover, MA)

[2] Purchased from Wellington Laboratories (Guelph, ON)

Table S2. List of PAHs names and abbreviations as well as data for GC-MS analysis such as retention time, ions monitored, surrogate standards used for quantitation and calibration information. Footnote on compound name indicates standard manufacturer.

Compound Name	Abbr.	Approx. Retention Time (min)	Quantitative Ion	Qualitative Ions	Quantitation Standard	Linear Range (pg µL^{-1})	R^2
d_{10}-Fluorene[1]	dFLU	15.6	176	174, 177	dBeP	---	---
Fluorene[2]	FLU	15.6	166	165	dFLU	3.8-1284	0.999
d_{10}-Phenanthrene[1]	dPHEN	18.2	188	160	dBeP	---	---
Phenanthrene[2]	PHEN	18.2	178	152, 176	dPHEN	3.8-1284	0.999
d_{10}-Anthracene[1]	dANT	18.3	188	160	dBeP	---	---
Anthracene[2]	ANT	18.4	178	125, 176	dANT	3.8-1284	0.999
d_{10}-Fluoranthene[1]	dFL	21.4	212	208	dBeP	---	---
Fluoranthene[2]	FL	21.4	202	101, 200	dFL	3.8-1284	0.999
d_{10}-Pyrene[1]	dPYR	21.9	212	106, 211	dBeP	---	---
Pyrene[2]	PYR	22.0	202	101, 201	dPYR	3.8-1284	0.999
d_{12}-Benz(a)anthracene[1]	dBaA	25.5	240	241	dBeP	---	---
Benz(a)anthracene[2]	BaA	25.5	228	114, 226	dBaA	3.8-1284	0.999
d_{12}-Chrysene[1]	dCHY	25.6	240	241	dBeP	---	---
Chrysene[2]	CHY	25.7	228	226, 227	dCHY	3.8-1284	0.999
d_{12}-Benzo(b)fluoranthene[1]	dBbF	29.3	264	132, 265	dBeP	---	---
Benzo(b)fluoranthene[2]	BbF	29.4	252	126, 251	dBbF	3.8-1284	0.999
d_{12}-Benzo(k)fluoroanthene[1]	dBkF	29.4	264	132, 265	dBeP	---	---
Benzo(k)fluoroanthene[2]	BkF	29.5	252	126, 253	dBkF	3.8-1284	0.999
d_{12}-Benzo(e)pyrene[1]	dBeP	30.1	264	265,261	Internal Standard	---	---
d_{12}-Benzo(a)pyrene[1]	dBaP	30.3	264	132, 261	dBeP	---	---
Benzo(a)pyrene[2]	BaP	30.6	252	126, 253	dBaP	3.8-1284	0.999
d_{12}-Indeno(1,2,3-CD) pyrene[1]	dIND	35.2	288	144, 289	dBeP	---	---
Indeno(1,2,3-CD) pyrene[2]	IND	35.3	276	138, 274	dIND	3.8-1284	0.999
d_{14}-Dibenz(a,h)anthracene[1]	dDBA	35.3	292	144	dBeP	---	---
Dibenz(a,h)anthracene[2]	DBA	35.4	278	139, 274	dDBA	3.8-1284	0.999
d_{12}-Benzo(g,h,i)perylene[1]	dBghiP	36.4	288	285	dBeP	---	---
Benzo(g,h,i)perylene[2]	BghiP	36.5	276	138, 274	dBghiP	3.8-1284	0.999

[1] Purchased from Cambridge Isotope Laboratories (Andover, MA)

[2] Purchased from Accustandard (New Haven, CT)

Table S3. Method validation data for each PAH and PAH surrogate including percent recovery from reproducibility study, method detection limit (MDL), and detected versus reported amounts from SRM 2585 with calculated percent error. Compound abbreviations are given in Table S2.

	Precision & Accuracy		SRM 2585		
**Compound Abbr.**	**Percent Recovery (n=6; %)**	**MDL (ppb)**	**Detected (pg mg^{-1})**	**Reported (pg mg^{-1})**	**% Error**
dFLU	67.9 ± 2.6	---	---	---	---
FLU	98.5 ± 1.3	14.5	483 ± 541	---	---
dPHEN	70.2 ± 3.0	---	---	---	---
PHEN	110 ± 4	85.7	2544 ± 427	1920 ± 20	32%
dANT	60.4 ± 2.4	---	---	---	---
ANT	100 ± 2	4.60	357 ± 398	96 ± 5.2	272%[1]
dFL	61.5 ± 2.1	---	---	---	---
FL	108 ± 1	26.0	4026 ± 223	4380 ± 100	8%
dPYR	65.0 ± 2.1	---	---	---	---
PYR	104 ± 1	18.0	3205 ± 276	3290 ± 30	3%
dBaA	79.7 ± 3.1	---	---	---	---
BaA	102 ± 1	3.07	1121 ± 94	1160 ± 54	3%
dCHY	67.3 ± 1.8	---	---	---	---
CHY	105 ± 1	4.95	2805 ± 129	2260 ± 60	24%
dBbF	82.7 ± 4.9	---	---	---	---
BbF	90.8 ± 3.4	19.3	3510 ± 192	2700 ± 90	30%
dBkF	64.9 ± 2.1	---	---	---	---
BkF	105 ± 4	5.61	1213 ± 116	1330 ± 70	9%
dBaP	76.6 ± 2.5	---	---	---	---
BaP	99.5 ± 1.3	8.28	947 ± 149	1140 ± 10	17%
dIND	76.6 ± 3.4	---	---	---	---
IND	104 ± 4	9.56	2356 ± 301	2080 ± 100	13%
dDBA	74.7 ± 3.3	---	---	---	---
DBA	107 ± 1	3.48	449 ± 183	301 ± 50	49%
dBghiP	76.3 ± 3.5	---	---	---	---
BghiP	104 ± 1	4.63	2095 ± 229	2280 ± 40	8%

[1] Detected and reported concentrations were near ANT method detection limits, which may explain the large discrepancy not observed in any other PAHS.

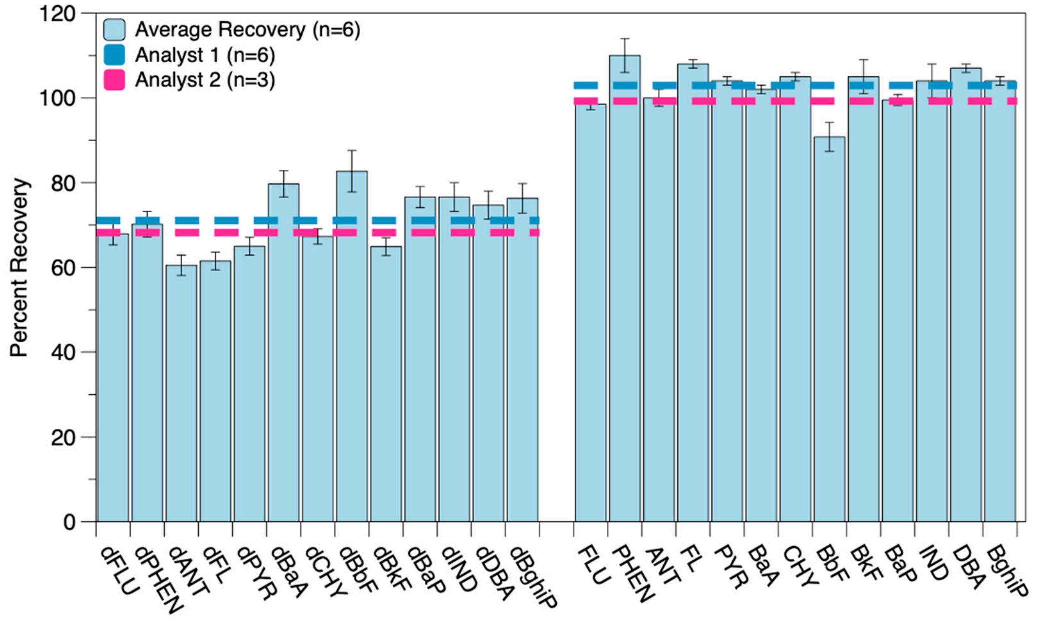

Figure S1. Average recoveries from Analyst 1's reproducibility study (n = 6) of the final analytical method for PAHs. The left set blue bars represent average surrogate recoveries while the right set of blue bars indicate surrogate- and blank-corrected target analyte recoveries. The overall average recoveries for this study are indicated by the dark blue dashed line. The overall average surrogate and target analyte recoveries from Analyst 2's reproducibility study (n = 3) are indicated by the dashed pink like. Compound names and abbreviations are given in Table S2.

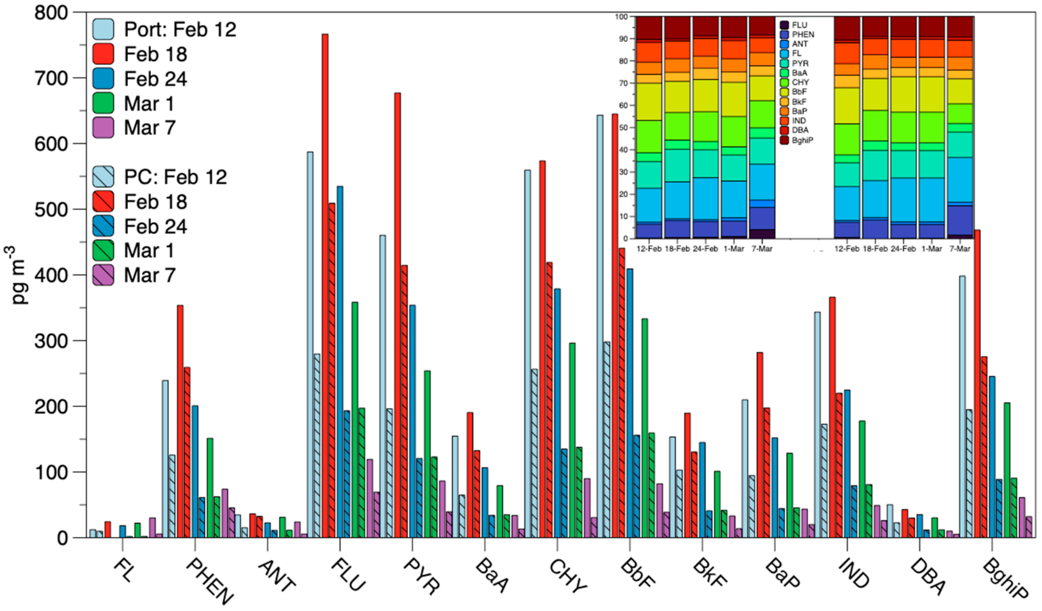

Figure S2. Atmospheric particulate matter concentrations of detected PAHs in samples taken at the Port of Providence (solid bars) and Providence College (PC; striped bars). Inlay shows percent composition of ΣPAH in each sample for each compound and suggests consistent sources of PAHs at each site, despite Port concentrations being consistently higher than PC concentrations. This bears further investigation. Compound names and abbreviations are given in Table S2.
